# The challenge of choosing in cardiovascular risk management

**DOI:** 10.1007/s12471-021-01599-y

**Published:** 2021-07-14

**Authors:** R. M. Hoogeveen, N. M. J. Hanssen, J. R. Brouwer, A. Mosterd, C. J. Tack, A. A. Kroon, G. J. de Borst, J. ten Berg, T. van Trier, J. Roeters van Lennep, A. Liem, E. Serné, F. L. J. Visseren, J. H. Cornel, R. J. G. Peters, J. W. Jukema, E. S. G. Stroes, the PANORAMA working group

**Affiliations:** 1 Department of Vascular Medicine, Amsterdam University Medical Centres, location AMC, Amsterdam, The Netherlands; 2 Medcon International, Heemstede, The Netherlands; 3 Department of Cardiology, Meander Medical Centre, Amersfoort, The Netherlands; 4 Department of Internal Medicine, University Medical Centre Nijmegen, Nijmegen, The Netherlands; 5 Department of Internal Medicine and Cardiovascular Research Institute Maastricht (CARIM), Maastricht University Medical Centre (MUMC+), Maastricht, The Netherlands; 6 Department of Vascular Surgery, University Medical Centre Utrecht, Utrecht, The Netherlands; 7 Department of Cardiology, Sint-Antonius Ziekenhuis, Nieuwegein, The Netherlands; 8 Department of Cardiology, Amsterdam University Medical Centres, location AMC, Amsterdam, The Netherlands; 9 Department of Internal Medicine, Erasmus MC, University Medical Centre Rotterdam, Rotterdam, The Netherlands; 10 Department of Cardiology, Sint Franciscus Gasthuis, Rotterdam, The Netherlands; 11 Department of Vascular Medicine, Amsterdam University Medical Centres, location VUmc, Amsterdam, The Netherlands; 12 Department of Vascular Medicine, University Medical Centre Utrecht, Utrecht, The Netherlands; 13 Department of Cardiology, Radboud University Medical Centre, Nijmegen, The Netherlands; 14 Department of Cardiology, Northwest Clinics, Alkmaar, The Netherlands; 15 Department of Cardiology, Leiden University Medical Centre, Leiden, The Netherlands

**Keywords:** Cardiovascular disease, Cardiovascular risk management, Prevention, Atherosclerosis, Novel interventions, Drugs

## Abstract

Cardiovascular disease (CVD) is a major cause of morbidity and mortality worldwide. For many years guidelines have listed optimal preventive therapy. More recently, novel therapeutic options have broadened the options for state-of-the-art CV risk management (CVRM). In the majority of patients with CVD, risk lowering can be achieved by utilising standard preventive medication combined with lifestyle modifications. In a minority of patients, add-on therapies should be considered to further reduce the large residual CV risk. However, the choice of which drug combination to prescribe and in which patients has become increasingly complicated, and is dependent on both the absolute CV risk and the reason for the high risk. In this review, we discuss therapeutic decisions in CVRM, focusing on (1) the absolute CV risk of the patient and (2) the pros and cons of novel treatment options.

## Introduction

Cardiovascular disease (CVD) is a major cause of morbidity and mortality. Traditional risk factors associated with increased cardiovascular (CV) risk comprise age, sex, elevated cholesterol, high blood pressure, diabetes and a family history of early CVD, combined with lifestyle factors such as smoking, obesity and physical inactivity. Chronic inflammatory diseases such as rheumatoid arthritis are also hallmarked by an increased CVD risk. For many years, medication used in CVD management was restricted to a combination of aspirin, beta-blockers, angiotensin-converting-enzyme inhibition and statins. Consequently, therapeutic decisions were relatively straightforward. Despite this successful guideline-based regimen a high residual CV risk remains. Particularly in patients with polyvascular disease, recurrent CV events and diabetes, the annual on-treatment re-event risk may be as high as 2.5–5%. This burden of modifiable residual risk can be reduced by initiating novel therapeutic agents with proven CV benefit.

In the majority of patients, marked CV risk lowering can be achieved by optimising standard therapy, including lifestyle modification. For a minority of patients, more intensive therapeutic drug interventions are required to further reduce the residual CV risk. The advent of novel drugs with clear CV benefit has broadened the options for CV risk management (CVRM). Paradoxically, the abundance of novel treatment options has also complicated clinical decision-making. The different international CV guidelines are not aligned with respect to recommendations for the use of ‘novel’ therapeutic agents, which results in greatly diverse therapeutic decisions among healthcare practitioners. In this tombola, the specific specialisation or interest of the prescribing physician (e.g. general practitioner, internist or cardiologist) often prevails in the drug choice, while the background and absolute CV risk of a particular patient should ideally determine therapeutic decisions.

The choice as to which combination of drugs is likely to offer the largest CV risk reduction in a specific patient is increasingly complicated. Due to this complexity, novel methods to stratify risk are necessary to assist prescribing physicians in their decision-making process. Currently available risk prediction models approved by the European Society of Cardiology (ESC), including the ESC CVD Calculation Risk App and the U‑Prevent tool, do not yet provide the information necessary to guide clinical decisions. Awaiting further refined decision-support systems, it is worthwhile to delineate the current framework for optimal CV therapeutic regimens including the novel therapeutic agents.

In this review, we discuss therapeutic decisions in CVRM, focusing on (1) the absolute CV risk of the patient and (2) the pros and cons of novel treatment options. We emphasise that, as with all models, this framework represents a simplification of reality. Moreover, it should be noted that physicians always have to take into account specific patient characteristics which cannot be entered in calculators, for instance comorbidity including pregnancy, family history or frailty of a patient entering the consulting room. Hence, clinical judgement is likely to remain essential.

## The absolute risk of the patient

Prior to embarking on the choice of specific therapeutic agents, it is essential to first address the absolute CV risk of a patient. The reason for this is that the absolute risk reduction achieved by any intervention is highly dependent on the absolute ‘baseline’ CV risk of a patient, making it crucial to carefully assess the absolute CV risk in each individual patient. According to current Dutch CVRM guidelines, risk should be assessed by allocating a subject to a risk category (very high, high or moderate risk). If a subject does not fit one of the risk categories, the CVRM guideline recommends using the SCORE table. Subjects with pre-existing CVD are all assigned to the highest risk category. However, the recurrence risk may vary widely in patients with pre-existing CVD. In secondary prevention patients in the SMART cohort, one fifth of patients had a 10-year risk lower than 10%, while this risk exceeded 40% in one fifth of patients [1]. Therefore, it is imperative to carefully estimate the absolute risk of patients with CVD prior to deciding on the optimal CV therapy [2, 3]. Although the most recent CV guidelines (ESC/European Atherosclerosis Society (EAS) [4]) do not recommend routine assessment of absolute CV risk in secondary prevention patients, the number of risk categories in these guidelines has increased (now including patients at very high and/or extremely high risk) and refer to the possibility of assessing absolute CV risk in several patient populations, including secondary prevention patients.

Absolute risk should, of course, be considered in light of competing risks, overall quality of life as well as the frailty of a specific patient. In patients with a shorter life expectancy, other treatment choices may apply than for fitter patients with fewer competing risks. Ideally, therapeutic options and patient preferences should be discussed via shared decision-making.

## Choice of interventions

**Fig. 1:**
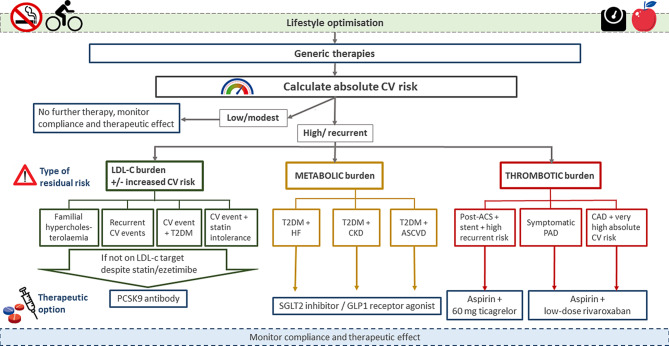
Assigning patients to subcategory boxes for optimised ‘novel’ therapies. *CV* cardiovascular, *LDL‑C* low-density lipoprotein cholesterol, *T2DM* type 2 diabetes mellitus, *HF* heart failure, *CKD* chronic kidney disease, *ASCVD* atherosclerotic cardiovascular disease, *ACS* acute coronary syndrome, *PAD* peripheral artery disease, *CAD* coronary artery disease, *PCSK9* proprotein convertase subtilisin/kexin type 9, *SGLT2* sodium-glucose cotransporter‑2, *GLP1* glucagon-like peptide‑1

In general, the absolute risk of patients can be attributed to a variety of risk factors. It is important to identify the origin of these major ‘residual’ risk factors in order to be able to choose the best therapeutic strategies. Here, we discuss targeting of residual lipid risk, residual thrombotic risk and residual risk due to diabetes and inflammation in patients with established CVD (Tab. 1, Fig. 1).

**Table 1:** Overall cardiovascular risk reduction in recent recent randomised controlled trials

Drug	CV risk reduction (HR, 95% CI)	Primary endpoint	Risks of drug use	Reimbursement criteria	Cost per patient per year^a^
Ezetimibe	0.94, 0.89–0.99 [9]	CV mortality, MI, unstable angina with hospitalisation, coronary revascularisation, stroke	Low	Primary and secondary prevention	~ €22–300
PCSK9i	0.85, 0.78–0.93 [2, 3]	Death from coronary heart disease, MI, stroke, unstable angina with hospitalisation, coronary re-vascularisation	Low	Familial hypercholesterolaemia and secondary prevention (conditions apply)	~ €3100–5600
SGLT2i	0.86, 0.80–0.93 [15]	CV mortality, MI, stroke	Low	T2DM (conditions apply)	~ €600–1200
GLP1-RA	0.90, 0.82–0.99 [25]	CV mortality, MI, stroke	Low	T2DM (conditions apply)	~ €1000–4600
DAPT	0.84, 0.74–0.95 [27]	CV mortality, MI, stroke	TIMI major bleeding: HR 2.32 (1.68–3.21)	Secondary prevention (post-ACS)	~ €60–1400
Low-dose rivaroxaban	0.84, 0.72–0.97 [52]	CV mortality, MI, stroke	TIMI major bleeding: HR 3.46 (2.08–5.77)	Secondary prevention for coronary heart disease or peripheral arterial disease	~ €3500
Low-dose colchicine	0.77, 0.61–0.96 [60]	CV mortality, reanimation, MI, stroke and coronary revascularisation	Higher risk of pneumonia	Gout, familial Mediterranean fever	~ €50–130

*HR* hazard ratio, *CI* confidence interval, *CV* cardiovascular, *MI* myocardial infarction, *PCSK9i* proprotein convertase subtilisin/kexin type 9 inhibitor, *SGLT2i* sodium-glucose cotransporter‑2 inhibitor, *T2DM* type 2 diabetes mellitus, *GLP1-RA* glucagon-like peptide‑1 receptor agonist, *DAPT* dual antiplatelet therapy, *TIMI* thrombolysis in myocardial infarction, *ACS* acute coronary syndrome

^a^Based on data found on www.medicijnkosten.nl, accessed 16 June 2020

### Residual low-density lipoprotein cholesterol risk

It is well established that a high level of low-density lipoprotein cholesterol (LDL-C) is a causal risk factor for CVD. Independent of the agents used, lowering of LDL‑C results in a reduction of CV events without a lower limit for LDL‑C beyond which the clinical benefit is lost. Early hints that very low LDL‑C levels were associated with impaired cognitive function could not be confirmed in subsequent large randomised clinical trials using either statin/ezetimibe combination therapy [5] or proprotein convertase subtilisin/kexin type 9 inhibitors (PCSK9i) [6, 7], further substantiating the safety of highly-potent LDL-C-lowering therapies. Collectively, these data have shifted the focus from target-based LDL‑C lowering to the goal of eradicating LDL‑C for optimal CV risk reduction. The degree of acceptation of this concept varies widely between guidelines. The Dutch CVRM guidelines advocate an LDL‑C target < 1.8 mmol/l in patients with established CVD and < 2.6 mmol/l in patients at increased risk for CVD. Conversely, the 2019 ESC/EAS guidelines introduced a variable LDL‑C target level dependent on the absolute CV risk of a patient. In patients with a very high CV risk LDL-C < 1.4 mmol/l is recommended, and for patients at extremely high risk an LDL-C < 1.0 mmol/l.

With respect to the choice of medication, statins remain the cornerstone for LDL‑C lowering. In patients in whom the guideline-recommended LDL‑C level is not achieved, ezetimibe should be added. Ezetimibe is generically available at low cost and lowers LDL‑C by approximately 20%, irrespective of the background therapy. If LDL‑C targets are not achieved by the combination of statin and ezetimibe, addition of a PCSK9i should be considered in very high CVD risk patients. PCSK9 antibodies are fully human, monoclonal antibodies that bind selectively to the PCSK9 protein. Sequestering PCSK9 increases the availability of LDL receptors on the liver cell surface, resulting in increased LDL‑C clearance from the circulation, leading to an additional LDL‑C lowering of 60%. Outcome trials have substantiated the CV benefit of PCSK9i, leading to a reduction in the risk of major adverse CV events (MACE) in two large CV outcome trials in 46,488 patients (hazard ratio (HR) 0.85, 95% confidence interval (CI) 0.79–0.92 [8] and HR 0.85, 95% CI 0.78–0.93) [9].

In the Netherlands, PCSK9i are currently reimbursed in subjects with elevated CV risk who do not reach LDL‑C targets on maximum tolerated statin combined with ezetimibe. Elevated CV risk is defined as (1) familial hypercholesterolaemia, (2) a recurrent CV event, (3) type 2 diabetes mellitus (T2DM) with a CV event or (4) CV event with documented intolerance to at least three statins (one of them at the lowest dose).

PCSK9i are generally well tolerated, with reported side effects restricted to mild injection site reactions and complaints of nasopharyngitis. Due to the relatively high costs, PCSK9i are considered to be cost-effective in patients with a very high CV risk and specific patients at high risk, such as those with familial hypercholesterolaemia [4].

With the advent of novel therapeutic platforms, such as RNA-based therapies, a 50% reduction of LDL‑C can be achieved with only two injections per year [10]. Pending long-term efficacy and safety data of this intervention (ORION-4 study; NCT03705234), the small interfering RNA-based approach is expected to improve adherence and reduce the costs of treatment. The use of the RNA-based therapeutic platforms also allows effective targeting of other atherogenic lipid targets, such as lipoprotein(a) (Lp(a)) [11]. Following a promising phase II study using the N‑acetylgalactosamine (GalNAc) antisense drug to target apolipoprotein A (apoA) [12, 13], the outcome study is currently ongoing (HORIZON study; NCT04023552).

### Residual triglyceride risk

Large epidemiological as well as genetic studies have firmly established that triglyceride levels are independently associated with CVD risk [14, 15].

Recent studies suggest that the majority of the CV risk of triglyceride-rich particles is in fact conveyed by the cholesterol load in these particles. In genetic studies, Ference et al. showed that the CV risk is proportional to the change in apoB concentration, independent of whether the apoB is located on LDL‑C or a triglyceride-rich particle [16]. However, unlike LDL-C-lowering trials, which all showed a CV benefit, trials aimed at lowering triglycerides using fibrates failed to reveal a consistent reduction of CVD [17]. Recently, the results of the Reduction of Cardiovascular Events with Icosapent Ethyl Intervention Trial (REDUCE-IT) [18] reported a major CV benefit of a total daily dose of 4 g of the omega‑3 fatty acid icosapent ethyl in patients with a high CV risk and elevated baseline triglycerides, in addition to statin therapy. The primary efficacy endpoint (time to first occurrence of CV death, non-fatal myocardial infarction (MI), non-fatal stroke, coronary revascularisation or unstable angina) was reduced significantly (HR 0.75, 95% CI 0.68–0.83). Unfortunately, the limited TG-lowering effect, combined with a poorly chosen placebo (mineral oil) complicated evaluation of the true CV outcome effects of icosapent ethyl [19]. In fact, the announcement of a negative outcome of the STRENGTH study (4 g eicosapentaenoic acid (EPA)/docosahexaenoic acid (DHA); NCT02104817) has ignited a debate as to whether the outcome of the REDUCE-IT study is related to a triglyceride-lowering effect, or reflects other pleiotropic effects of high-dose EPA. To resolve this discussion, the results of the PROMINENT study (NCT03071692) are awaited, wherein the effect of a selective peroxisome proliferator-activated receptor (PPAR)-alpha modulator is evaluated in T2DM patients with combined dyslipidaemia; the results are expected in 2024.

### Residual metabolic risk (diabetes)

T2DM is a strong risk factor for CVD. Most glucose-lowering agents (sulphonyl urea (SU) derivatives, acarbose, dipeptidyl peptidase 4 (DDP4) inhibitors and insulin) do not seem to reduce the incidence of CV events apart from the potential benefit of better glucose control. Metformin is the traditional glucose-lowering agent that most convincingly lowers the risk of MI [20]. More recent studies with sodium-glucose cotransporter‑2 inhibitors (SGLT2i) and glucagon-like peptide‑1 receptor agonists (GLP-1RA) have demonstrated a robust CV benefit, seemingly unrelated to glycaemic regulation. The benefits seen with SGLT2 inhibitors are most likely derived through a reduction of heart failure (HF)-related endpoints, whereas the benefits seen with GLP1-RA most likely reflect a reduction of atherosclerosis-related events.

#### SGLT2 inhibitors

SGLT2i act via selective and reversible blocking of the SGLT2 in the proximal tubules in the kidney. This leads to glycosuria, resulting in lower blood glucose levels. Several large clinical trials with a total of 42,568 patients with T2DM and a high CV risk have shown that SGLT2i positively affect MACE, hospitalisation for HF and the mortality risk in patients with established CVD. A 14% reduction in MACE was observed in the EMPA-REG outcome trial and the CANVAS programme [21, 22], although DECLARE TIMI 58 and the VERTIS CV trial failed to show a reduction in MACE [23, 24]. A 27–35% reduction in hospitalisations for HF has been substantiated across the SGLT2i class [25, 26]; this benefit was similarly observed in HF patients with or without T2DM [26, 27]. SGLT2i also reduce the risk of the composite renal outcome defined as 40–57% decrease in estimated glomerular filtration rate, end-stage kidney disease, or renal death (HR 0.55, 95% CI 0.48–0.64; *p* < 0.001) [28–30]. Finally, a 38% reduction in CV death was observed in the EMPA-REG [21]. In line with these findings, the latest ADA/EASD [31] as well as ESC/EASD [32] guidelines recommend SGLT2i as preferable therapy in T2DM patients when HF with reduced ejection fraction or chronic kidney disease predominates [31]. In the Netherlands, SGLT2i are currently reimbursed in patients with T2DM treated with oral glucose-lowering agents, but updated indications are expected. At present, there is a ‘pay-back’ arrangement for patients using insulin.

Side-effects of SGLT2i are generally mild and mainly consist of genital infections; however, there is a slightly higher risk of development of diabetic ketoacidosis. In contrast to other guidelines, the Dutch Association of General Practitioners (NHG) does not currently recommend the use of SGLT2i, although this view is currently being reconsidered.

#### GLP-1 receptor agonists

GLP-1RA activate the GLP‑1 receptors which are located throughout the body. Activation of GLP‑1 receptors in the islets of Langerhans results in increased insulin secretion and suppression of glucagon secretion, leading to reduction of the blood glucose concentration. GLP-1RA also result in a delay in gastric emptying and have a central appetite-lowering effect. GLP-1RA are hallmarked by a potent glucose-lowering effect, combined with weight loss and an anti-inflammatory effect [33, 34]. Although GLP-1RA are generally injectable therapies, for semaglutide an oral compound is also available.

Six CV outcome trials have been conducted with GLP-1RA, of which pooled data in 54,121 patients yielded an HR for CV mortality, non-fatal MI and non-fatal stroke of 0.90 (95% CI 0.82–0.99) in patients with CVD in non-inferiority trials [28, 35–40]. Differences between agents have been reported, whereby in contrast to exendin-4-based GLP‑1 molecules, human GLP‑1 analogues show a significant reduction in the relative risk for MACE of 26% for (subcutaneous) semaglutide, 22% for albiglutide, 13% for liraglutide and 12% for dulaglutide. CV mortality was reduced in several of the trials, by 22% [39] to 51% [41], whereas no benefit was observed regarding hospitalisation for HF [28]. In line with these findings, the latest American Diabetes Association (ADA)/European Association for the Study of Diabetes (EASD) [31] report recommends GLP-1RA with proven CV benefit as a preferable therapy in T2DM when a high atherosclerotic CV risk predominates.

Upcoming trials with GLP-1RA will also address the effect of GLP-1RA in non-diabetic patients with overweight/obesity and prior CV disease (SELECT; NCT03574597). The side effects of GLP-1RA are mostly gastrointestinal complaints. In the Netherlands, GLP-1RA is reimbursed for patients with T2DM and a body mass index ≥ 30 kg/m^2^ in combination with metformin and SU derivatives, or in combination with metformin and suboptimal treatment despite optimal basal insulin therapy (≥ 3 months).

### Residual thrombotic risk

In contrast to the aforementioned therapeutic interventions, targeting of thrombotic risk involves a delicate balance between the benefit of reducing the CVD risk and the risk of (major) bleeding. Every intervention that reduces the thrombotic risk also confers an increased bleeding risk. Irrespective of how much experience a physician has in prescribing antithrombotic therapy, assessing the thrombotic and bleeding risk may be challenging. Bleeding scores are available [42, 43], but are far from perfect in predicting the bleeding risk. Considering the inherent bleeding risk of antithrombotic medication, a prudent approach is crucial.

#### Dual anti-platelet therapy

In patients following a percutaneous coronary intervention (PCI) for acute coronary syndrome, dual antiplatelet therapy (DAPT) consisting of a potent P2Y12 receptor inhibitor in addition to aspirin is recommended for 12 months [44]. Shortening the duration of DAPT can be considered based on careful weighing up of the balance between absolute CV risk and individual bleeding risk [44]. A switch from potent P2Y12 receptor inhibition (prasugrel or ticagrelor) to less potent inhibitors (clopidogrel) can be considered as alternative treatment in patients with a markedly increased bleeding risk, taking into account that this heralds a potential for increased ischaemic risk particularly if performed early after PCI [45]. In CV patients with a very high (thrombotic) risk but no major bleeding risk, prolonged DAPT (ticagrelor; at a reduced dose of 60 mg b.i. d.) for up to 3 years on top of aspirin can be considered [46, 47]. This regimen was shown to reduce the CV risk (CV death/MI/stroke; HR 0.85, 95% CI 0.74–0.96), at the expense of a higher bleeding risk (TIMI major bleed; HR 2.32, 95% CI 1.68–3.21). In parallel, DAPT comprising ticagrelor and aspirin also reduced the incidence of MACE in patients with peripheral artery disease (PAD) and a prior MI [48], but not in PAD patients after peripheral revascularisation [49].

Yearly evaluation of the bleeding risk is needed before routinely prolonging DAPT after the first year. This is underscored by recent studies amongst patients after PCI, revealing that ticagrelor monotherapy after 3 months of DAPT was associated with a lower risk of bleeding, without a higher risk of CV events [50, 51].

#### Dual pathway inhibition

Rivaroxaban is an oral anticoagulant that directly inhibits factor Xa, inhibiting the plasma coagulation cascade. In the COMPASS trial [52] (27,395 patients with stable atherosclerotic disease), the addition of low-dose rivaroxaban (2.5 mg twice daily) to aspirin resulted in a significant CV risk reduction (HR 0.76, 95% CI 0.66–0.86), but at the expense of a significant increase in bleeding complications (HR 1.70, 95% CI 1.40–2.05). Effects were predominantly driven by a reduction of stroke incidence. Previously, rivaroxaban on top of clopidogrel has also been studied in patients with acute coronary syndrome, showing a reduction of ischaemic events and CV mortality along with a higher risk of bleeding [53]. Since data on the use of rivaroxaban plus potent P2Y12 receptor inhibitors are lacking, these data cannot be extrapolated to current practice. More recently, the effect of low-dose rivaroxaban on top of aspirin was also studied in patients with PAD following peripheral revascularisation. The composite efficacy outcome (acute limb ischaemia, amputation, MI, stroke or CV death) was reduced (HR 0.85, 95% CI 0.76–0.96), at the expense of an increased bleeding risk (HR 1.43, 95% CI 1.10–1.84) [54].

Collectively, these data imply that low-dose rivaroxaban (2.5 mg twice daily) on top of aspirin can be considered in patients with a very high CV risk and a high thrombotic risk, without an increased risk for major bleeding. The Dutch guidelines for PAD recommend clopidogrel as first-line therapy [55]; the 2019 ESC/EAS guidelines recommend considering the use of low-dose rivaroxaban in combination with aspirin for patients with diabetes and PAD and low bleeding risk [32]. In the Netherlands rivaroxaban is reimbursed for patients with coronary artery disease and symptomatic PAD who are treated with aspirin with or without clopidogrel, without a previous transient ischaemic attack or stroke. In patients with stable CVD, estimation of an absolute CV risk score ≥ 20% can also be considered as a threshold to start additional antithrombotic treatment.

### Residual inflammatory risk

Chronic low-grade inflammation plays an important role in all stages of atherosclerosis [56]. The CANTOS trial has proved the validity of targeting inflammation in CVD using the interleukin-1β monoclonal antibody canakinumab, showing a reduction in the incidence of CV events (HR 0.85, 95% CI 0.76–0.96), but also an (albeit small) increase in fatal infections [57]. However, not all strategies targeting inflammation have a beneficial effect in CVD (e.g. methotrexate) [58].

#### Colchicine

Colchicine is a cheap, mild anti-inflammatory drug that is used for prevention of gout, treatment of familial Mediterranean fever and pericarditis. Adding low-dose colchicine to standard treatment of patients with MI within the past 30 days (*n* = 4745) resulted in a significant CV risk reduction (HR 0.77, 95% CI 0.61–0.96) in the COLCOT trial [59]. The CV risk reduction was driven by a reduction in the number of urgent hospitalisations for revascularisation for angina pectoris and stroke. Colchicine had no effect on CV death or MI after a median follow-up of 22.6 months. Treatment with colchicine increased the risk of pneumonia (0.4% in the placebo group vs 0.9% in the colchicine group), but there was no increase in the risk of fatal infections [59]. The LoDoCo2 trial evaluated the effect of colchicine in 5522 patients with stable coronary heart disease [60]. The study showed that after a median follow-up of 28.6 months, patients randomised to low-dose colchicine (0.5 mg once daily) experienced fewer CV deaths, spontaneous (non-procedural) MI, ischaemic stroke or ischaemia-driven coronary revascularisation (HR 0.69, 95% CI 0.57–0.83) [60]. There was no sign of increased toxicity by combining colchicine with statin therapy. Thus, low-dose colchicine may be a valuable addition to further reduce the incidence of CV events in patients with both chronic, stable coronary heart disease and in those who have recently had an MI.

## Clinical implications

Clinicians are facing a large variety of novel medication options, which have all been shown to significantly reduce the residual CV risk in various patient categories. However, in contrast to cheap generic drugs, these novel treatment options are mostly expensive, necessitating careful selection of patients in order to ensure cost-effectiveness of these additional interventions. This new reality will impact on daily clinical practice. First, it is imperative to perform a reliable absolute risk assessment not only in primary but also in secondary prevention patients, since absolute risk is the major driver for absolute risk reduction following medical interventions. Second, the clinician needs to decide what ‘specific’ features do qualify patients for certain therapeutic strategies, which currently comprise intensive lipid lowering, metabolic agents or anticoagulant regimens (Fig. 1). Third, the clinician may also have to identify potential drugs, which might be discontinued without causing harm; albeit in the absence of randomised controlled trials validating these discontinuation decisions. As mentioned previously, standard therapy (medication + lifestyle) will suffice in the majority of patients. For the remainder of patients with a high but modifiable residual risk, novel treatments should be considered. Carefully weighing up the clinical features of the patient (like frailty, overweight, HF), laboratory values (lipids, inflammation) and the risk for adverse drug effects (e.g. bleeding risk) can assist in delineating the first choice for the add-on of novel therapy in patients (Fig. 1). Obviously, the patient’s personal preferences also have to be taken into account during shared decision-making to maximise therapy compliance. In very high risk patients, simultaneously targeting two types of risk may be the preferred strategy.

The oversimplified approach of assigning patients to subcategory boxes does not account for all aspects that should contribute to selecting ‘tailored’ therapy in patients. Moreover, not all patients are represented in the individual drug trials, hampering external validity of the data. Also, the order in which therapy is initiated has a large impact on the absolute benefit that can be achieved by a particular add-on intervention, since a lower baseline risk following ‘other therapies’ automatically reduces the residual risk and hence the absolute benefit conveyed by the subsequent agent. Finally, novel risk factors are continuously being identified in CVD, for which novel agents will eventually compete with the CV benefit of current ‘novel’ agents (for instance: Lp(a)-lowering, anti-inflammatory agents).

Taking all these aspects into account, the clinical horizon is likely to involve a decision-support system to help determine the choice as well as the ‘order’ of specific therapeutic interventions. These decision-support systems will have to be able to reliably calculate the net benefit of interventions, based on all available clinical trial data. Promising initiatives are currently emerging, among which are the lifetime risk and benefit calculators on www.U-Prevent.com and the ESC-CVD risk app (https://www.escardio.org/Education/ESC-Prevention-of-CVD-Programme/Risk-assessment/esc-cvd-risk-calculation-app). However, currently available models do have drawbacks, since they do not indicate the uncertainty of predictions (based on confidence intervals of the used trial data) and do not yet incorporate potential side effects of therapies. Calculators to estimate adverse effects of treatment, such as bleeding risk, have recently been developed. Eventually, decision-support tools need to weigh the net benefit versus the (potential) adverse effects of interventions. Cost-effectiveness and the preference of the patient (e.g. subcutaneous injections or daily oral drugs) will complement the final decision in a process of shared decision-making. Hurdles for computer-derived systems comprise a rigorous (regional) calibration of the underlying algorithms and the degree of automaticity of data acquisition in an effort to minimise time investment by the clinical staff.

## Conclusion

During the last decade, we have witnessed a large number of trials reporting a significant CV benefit of a wide variety of drugs. This positive development is hampered by the ensuing arbitrariness in choosing these novel add-on therapies, largely dependent upon the background of the specialist rather than a patient’s absolute CV risk. In this review, we have provided an overview and some ‘simplified’ handles allowing the physician to reach the best decision for individual CV risk reduction in specific categories of patients for whom standard therapy is insufficiently effective. We believe the box model of currently available treatment options (lipids, metabolic, anti-coagulant) can help optimise treatment in high-risk patients. The introduction of automated, computer-decision-support systems is awaited to reach the next level in tailored therapy in CV treatment of patients with a high but modifiable residual risk.
